# Supplementary figures and images for: Genomic and transcriptomic evidence for descent from Plasmodium and loss of blood schizogony in Hepatocystis parasites from naturally infected red colobus monkeys
Source: PLoS Pathog. 2020 Aug 3;16(8):e1008717. doi: 10.1371/journal.ppat.1008717 (PMC7425995; doi:10.1371/journal.ppat.1008717)

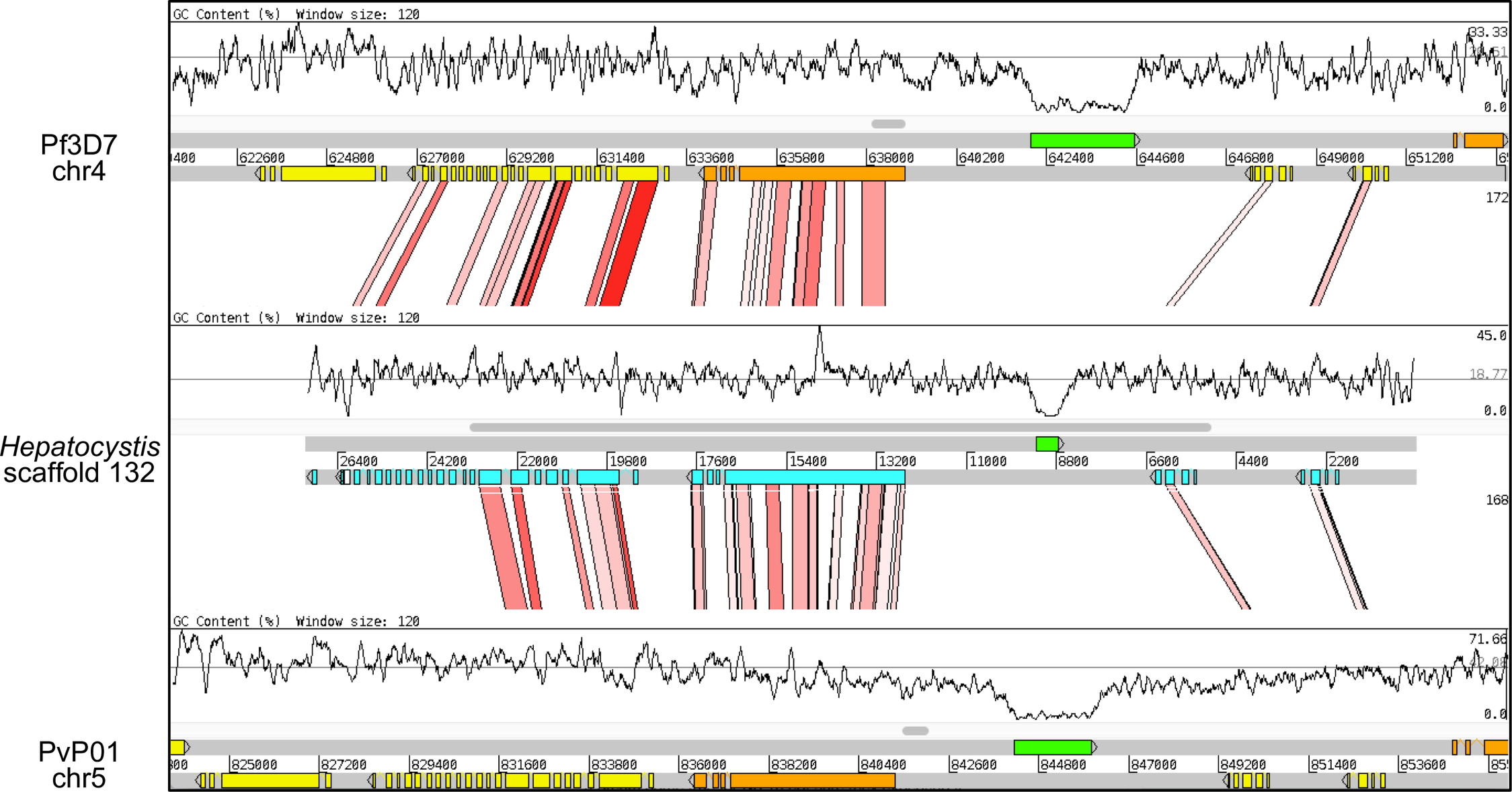

Supplement: S1 Fig — ACT (Artemis Comparison Tool) screenshot showing a comparison of centromere-proximal regions of Hepatocystis scaffold 132, P. falciparum 3D7 (Pf3D7) chromosome 4 and P. vivax (PvP01) chromosome 5. The red blocks represent sequence similarity (tBLASTx). The centromere is shown in green. Coloured boxes represent genes. The graph shows the GC-content. (TIF) [file ppat.1008717.s001.tif]

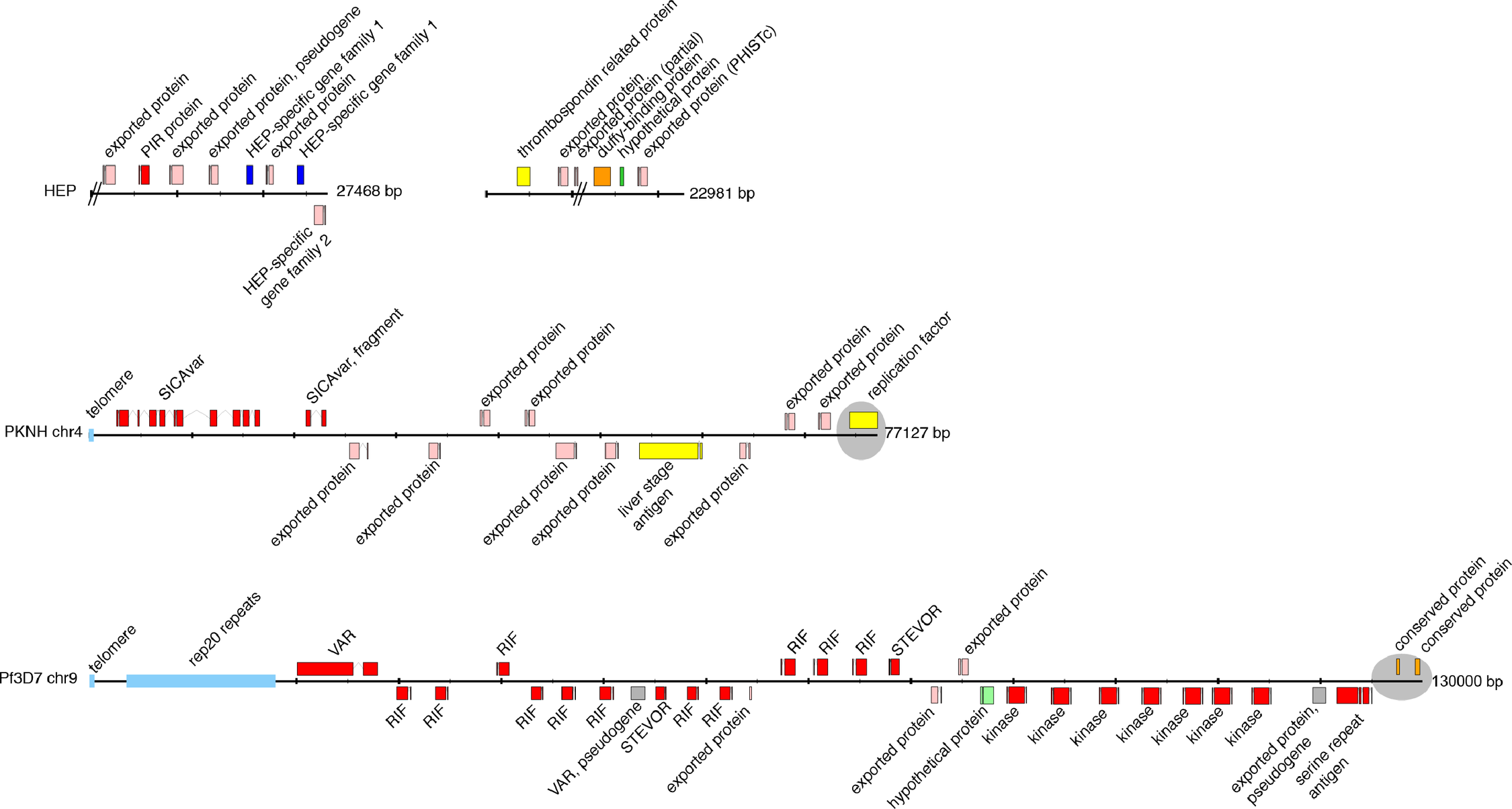

Supplement: S2 Fig — Exons are shown in coloured boxes with introns as linking lines. ‘//’ represents a gap. The shaded/grey areas in P. knowlesi and P. falciparum mark the start of the conserved, syntenic regions to other Plasmodium species. The presence of genes that are subtelomeric in Plasmodium species, i.e. PHIST proteins, suggests that the Hepatocystis scaffolds are also subtelomeric. A complete subtelomere that includes telomeric repeats is missing in our Hepatocystis assembly. Thus, whether Hepatocystis chromosomes retain the organisation common to most Plasmodium species remains unclear. (TIF) [file ppat.1008717.s002.tif]

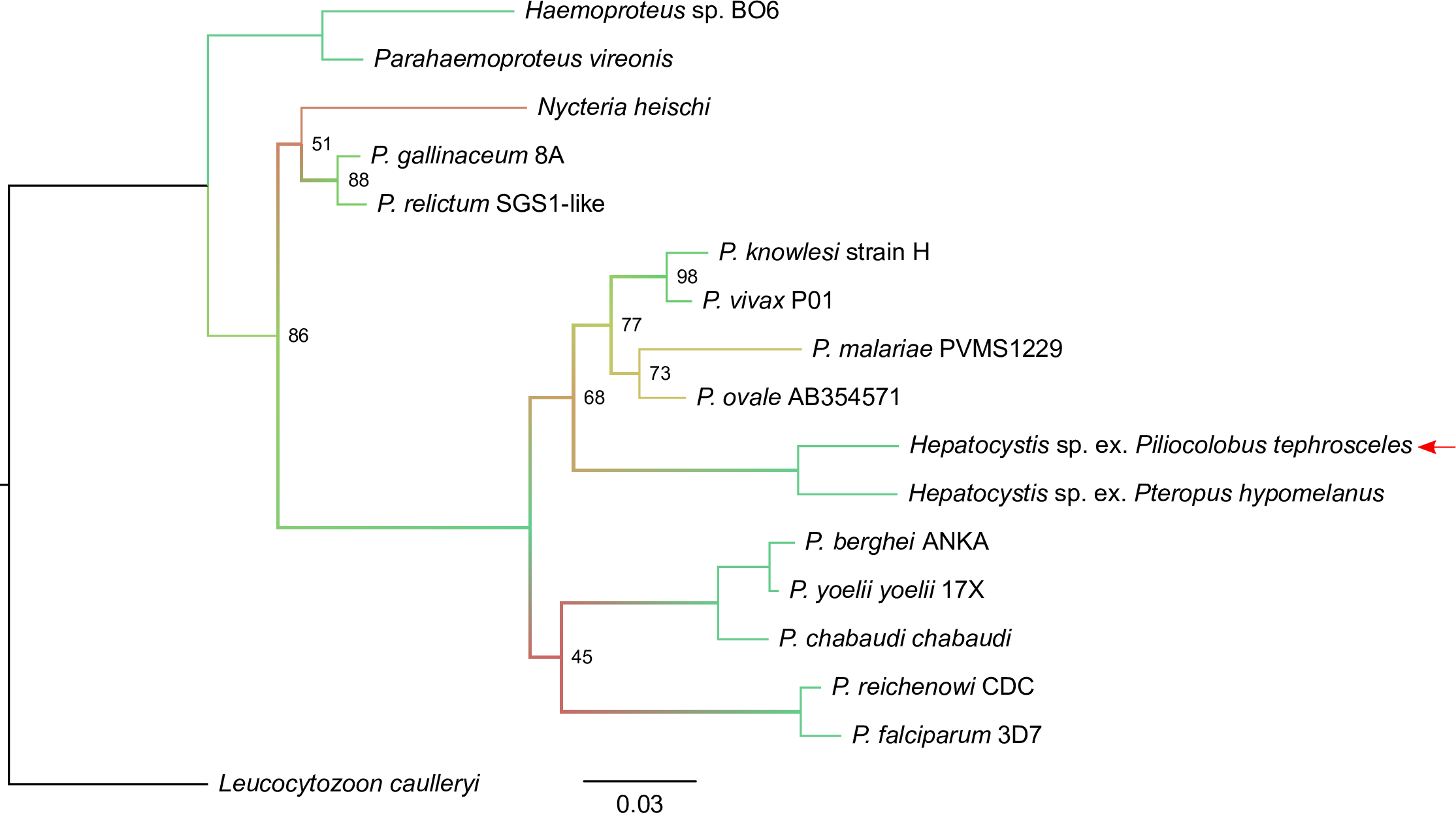

Supplement: S3 Fig — Hepatocystis sp. ex. Piliocolobus tephrosceles (this work, marked with red arrow) appears next to a previously sequenced Hepatocystis sample from the flying fox Pteropus hypomelanus (NCBI accession FJ168565.1). Branches of the tree have been coloured by bootstrap support values from 45 (red) to 100 (green). Bootstrap values below 100 have also been added to the figure as text. (TIF) [file ppat.1008717.s003.tif]

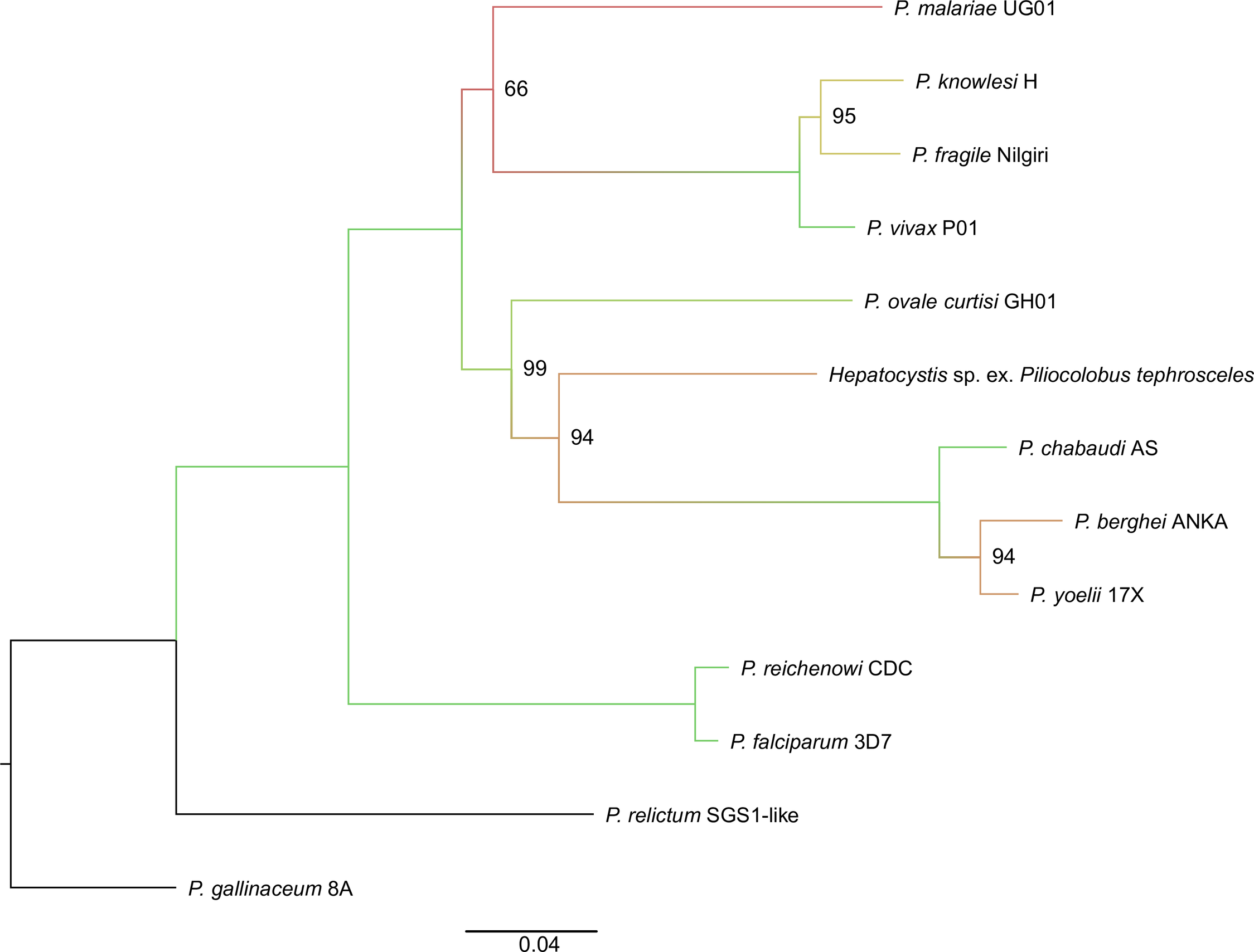

Supplement: S4 Fig — Branches of the tree have been coloured by bootstrap support values from 66 (red) to 100 (green). Bootstrap values below 100 have also been added to the figure as text. (TIF) [file ppat.1008717.s004.tif]

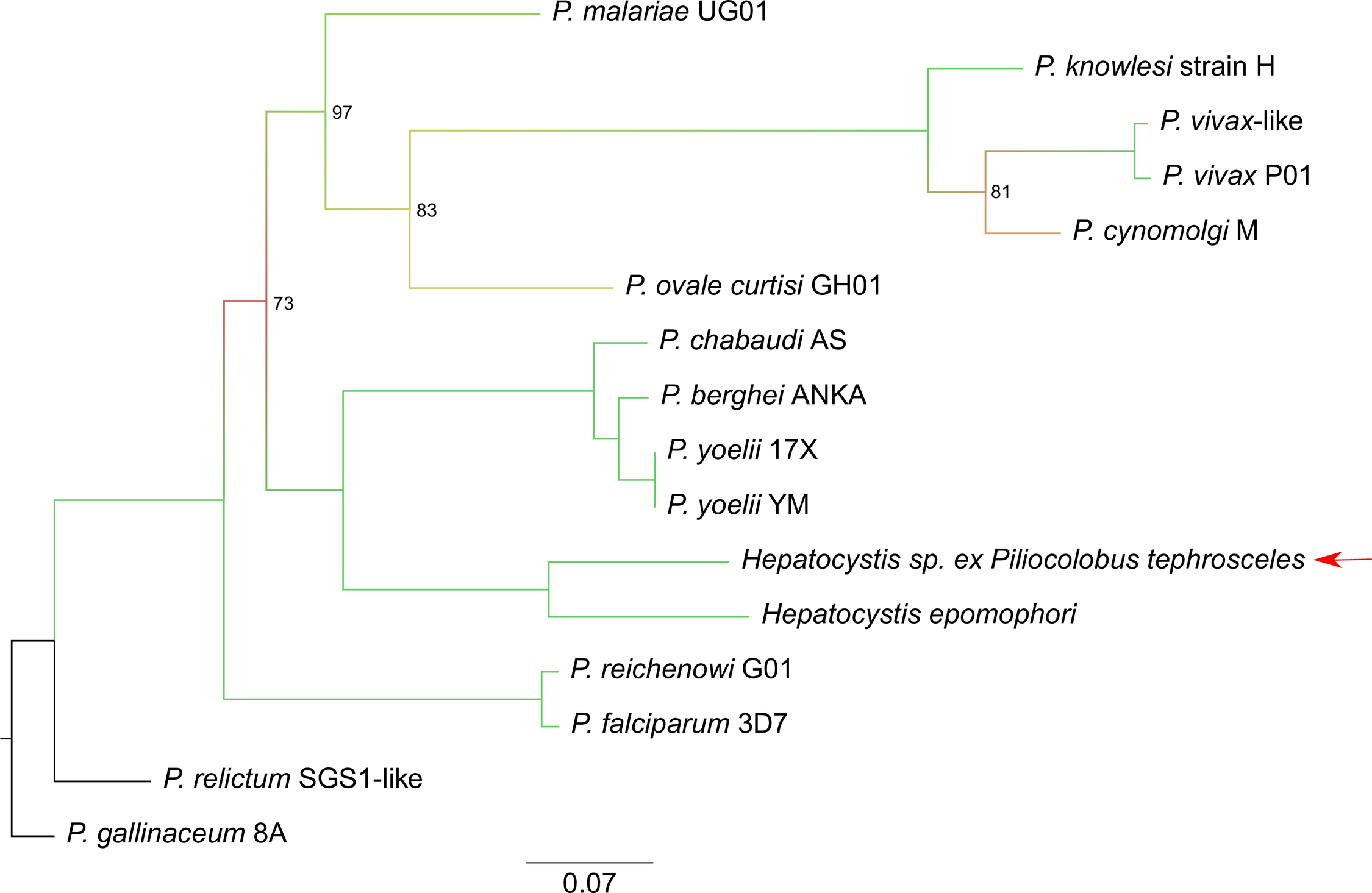

Supplement: S5 Fig — Genes of Hepatocystis sp. ex Piliocolobus tephrosceles are highly similar to Hepatocystis epomophori genes sequenced in a different study [2]. The tree is based on the following genes: splicing factor 3B subunit 1, tubulin gamma chain, DNA polymerase delta catalytic subunit, eukaryotic translation initiation factor 2 gamma subunit, T-complex protein 1 subunit alpha, pantothenate transporter, ribonucleoside-diphosphate reductase large subunit, aminophospholipid-transporting P-ATPase, GCN20, transport protein Sec24A and RuvB-like helicase 3. Branches of the tree have been coloured by bootstrap values from 73 (red) to 100 (green). Bootstrap values below 100 have also been added to the figure as text. The red arrow points to the Hepatocystis sample from the current study. (TIF) [file ppat.1008717.s005.tif]

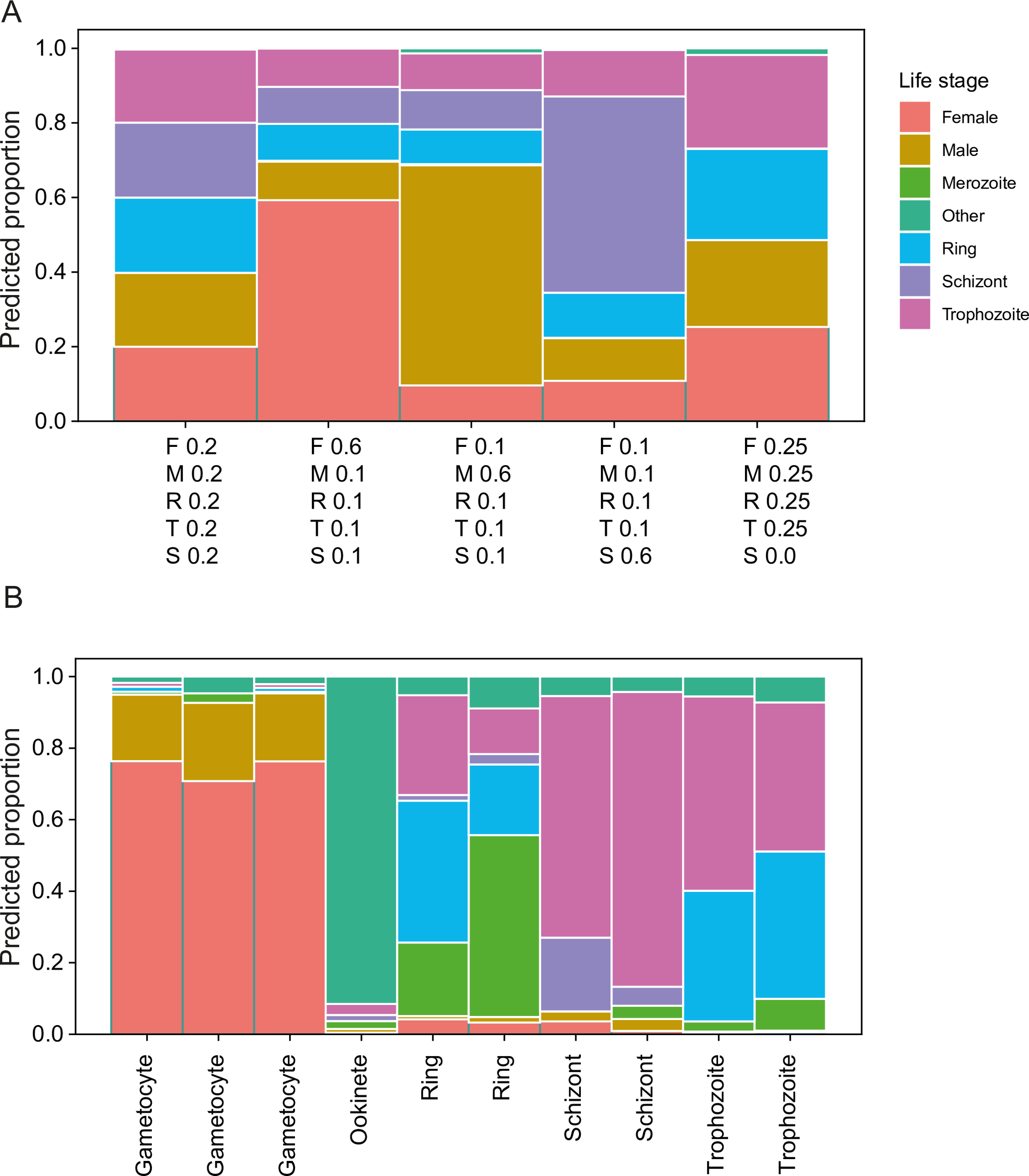

Supplement: S6 Fig — (A) Pre-defined mixtures of pseudobulk RNA-seq data were deconvoluted with very high accuracy. (B) Real samples of P. berghei bulk RNA-seq from Otto et al (2014) were deconvoluted showing almost pure mixtures of gametocyte, ookinete or asexual stages as expected. The low proportions of expected parts of the IDC in each asexual sample may result from differences between what the MCA defines as a ring/trophozoite/schizont and what would microscopically be defined as such. (TIF) [file ppat.1008717.s006.tif]

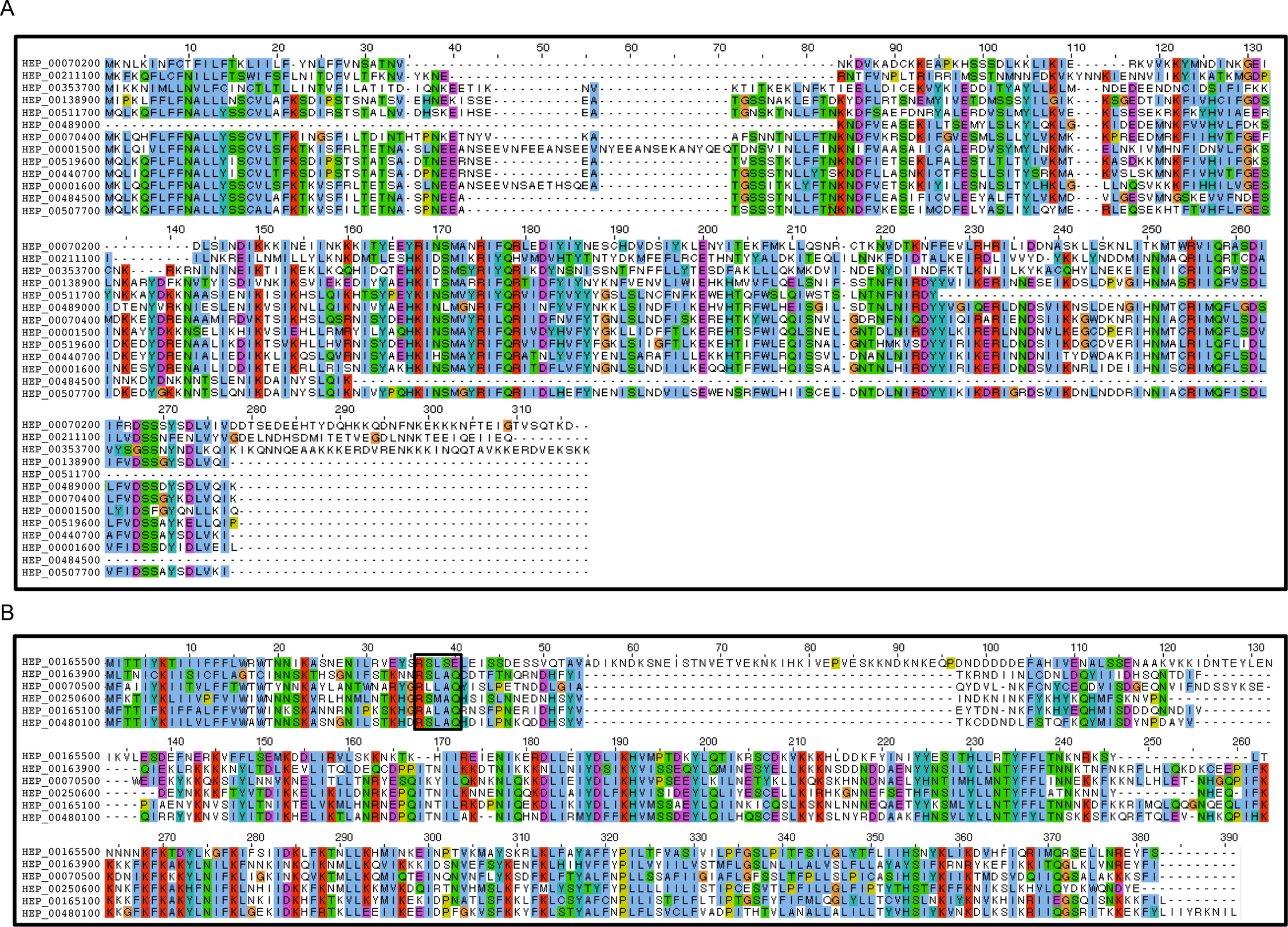

Supplement: S7 Fig — (A) Alignment of Hepatocystis-specific gene family 1 (hep1). Pseudogenes (HEP_00099300, HEP_00250500, HEP_00323900) were not included in the alignment. HEP_00353700 is 476 amino acids long and was truncated here. (B) Alignment of Hepatocystis-specific gene family 2 (Hep2). This gene family contains a PEXEL motif (marked with a black box). Pseudogenes (HEP_00165000, HEP_00165200, HEP_00324000, HEP_00489100) were not included in the alignment. (TIF) [file ppat.1008717.s007.tif]

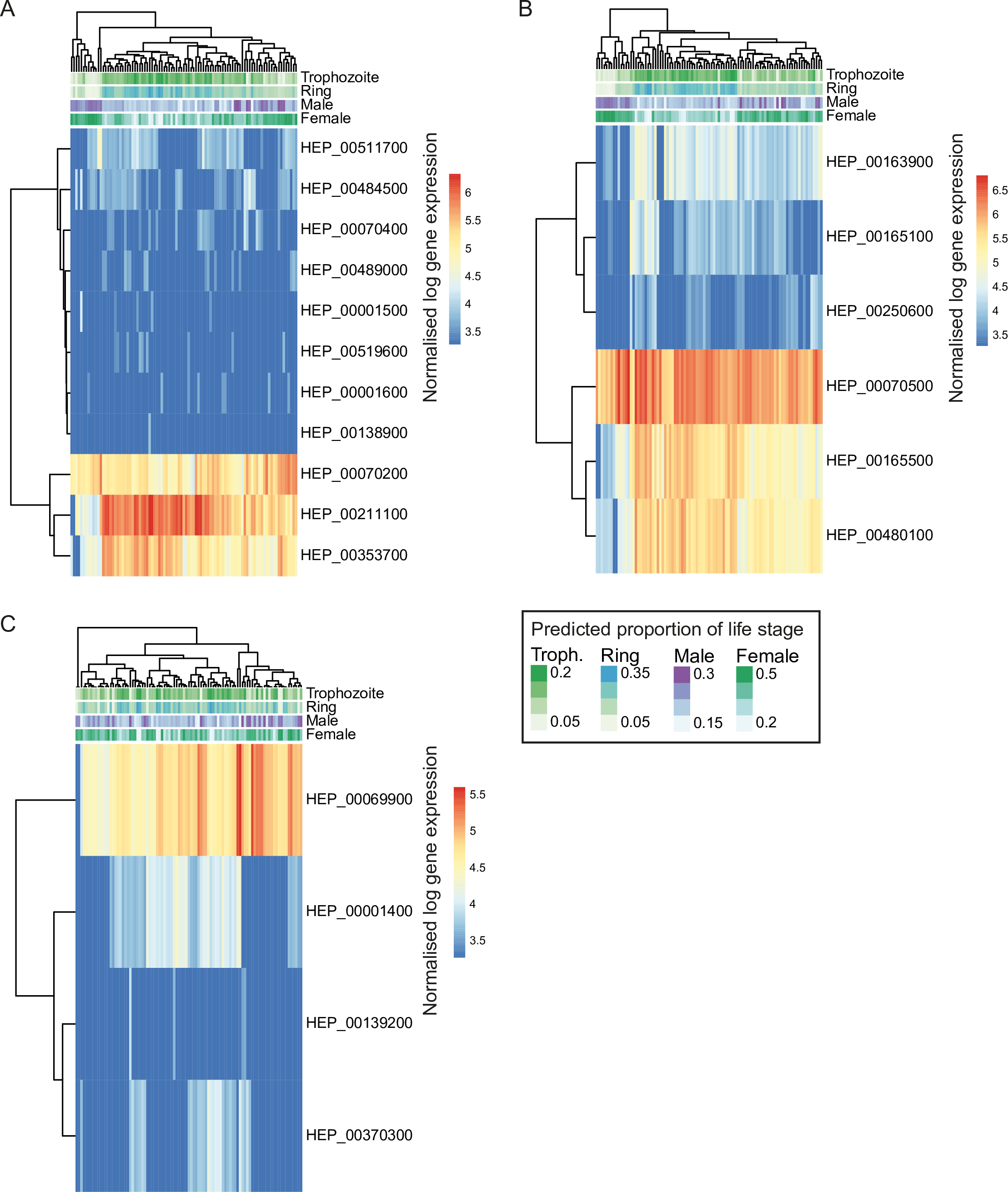

Supplement: S8 Fig — (A) Expression levels (log vst-normalised) of hep1 genes across blood samples from multiple red colobus monkeys. The estimated proportions of early blood stages (rings/trophozoites) and mature gametocytes are highlighted above. (B) Expression levels of hep2 genes (C) Expression levels of pir genes. (TIF) [file ppat.1008717.s008.tif]

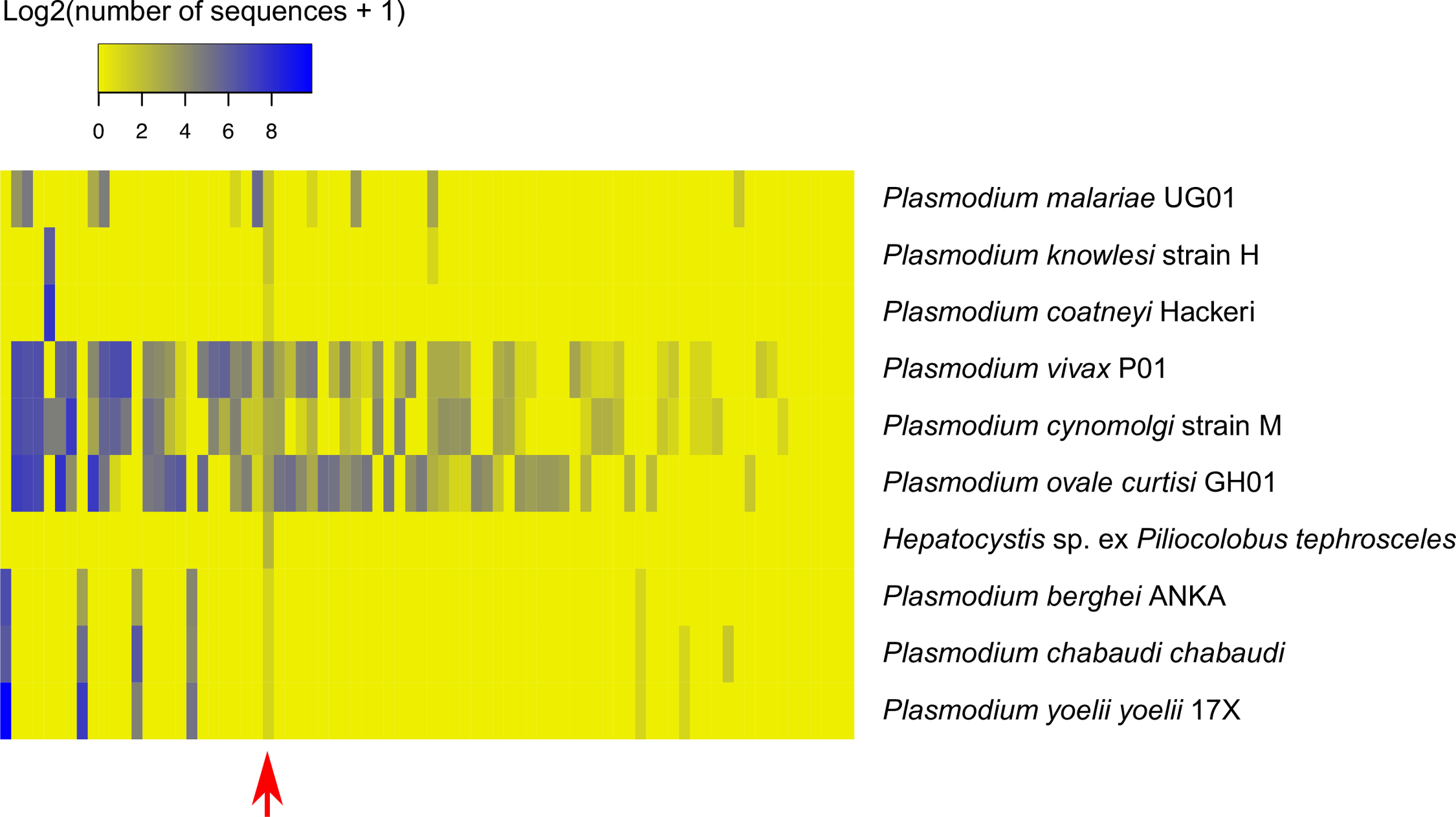

Supplement: S9 Fig — Rows correspond to species and columns correspond to pir subfamilies. The columns have been ordered by the number of sequences in each subfamily and the order of rows is approximately based on phylogeny. Colours represent the numbers of proteins belonging to each subfamily for each species. All Hepatocystis pir proteins belong to the only subfamily conserved across all these species [114] (indicated with red arrow). (TIF) [file ppat.1008717.s009.tif]

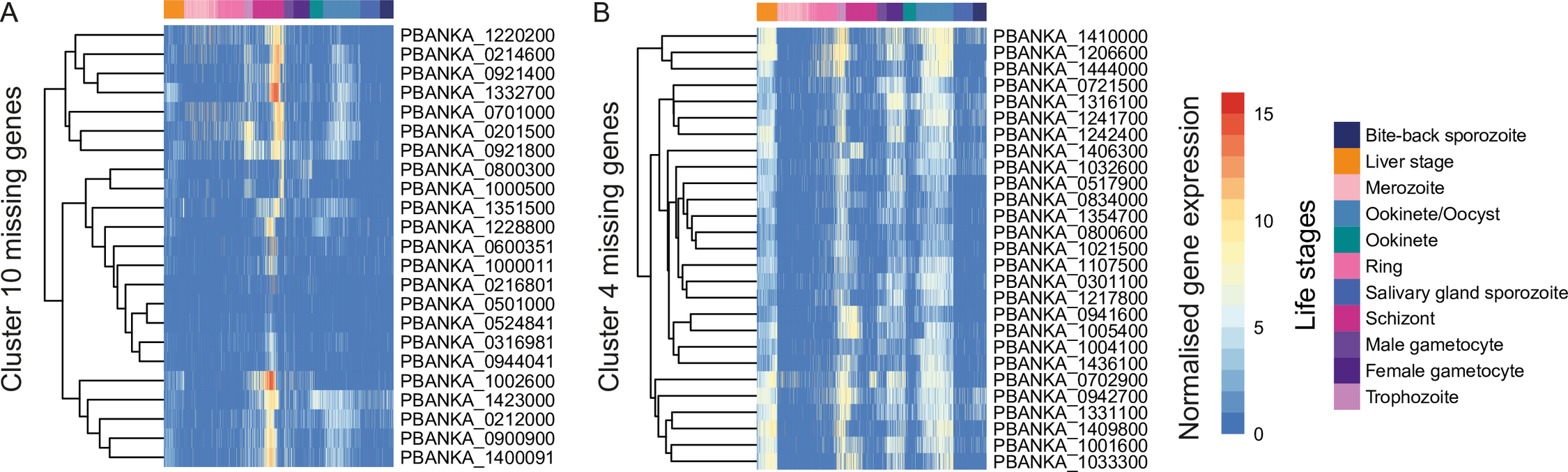

Supplement: S10 Fig — (A) Malaria Cell Atlas (MCA) gene cluster 10 represents genes highly expressed in late schizonts. 25 genes from this cluster were conserved in P. ovale wallikeri and P. vivax, but were missing from our Hepatocystis genome assembly. Genes were clustered here by expression pattern and single-cells were ordered by pseudotime as in [20]. (B) MCA cluster 4 represents genes highly expressed across much of the life cycle—liver stages, trophozoites, female gametocytes and ookinetes/oocysts. 27 genes from this cluster were conserved in P. ovale wallikeri and P. vivax, but were missing from our Hepatocystis genome assembly. (TIF) [file ppat.1008717.s010.tif]

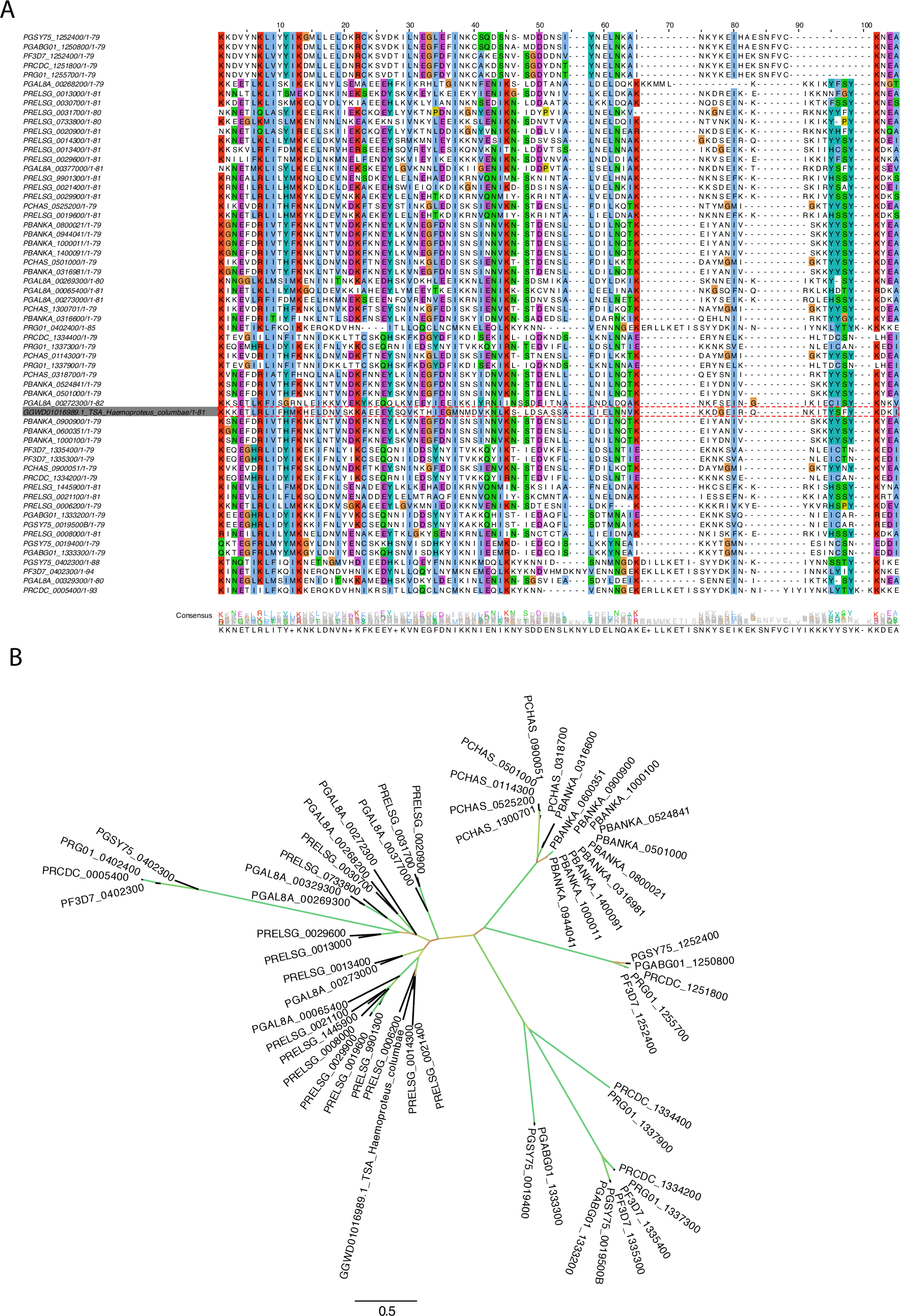

Supplement: S11 Fig — (A) Alignment of the translation of a sequence (GGWD01016989.1) from Haemoproteus columbae transcriptome assembly (GenBank: GGWD00000000.1) [23] with Plasmodium reticulocyte-binding proteins (RBPs) from PlasmoDB [115]. The alignment has been cropped to the length of the Haemoproteus columbae sequence. (B) Phylogenetic tree of Plasmodium RBPs and the Haemoproteus columbae sequence GGWD01016989.1, based on the alignment in panel A. Branch colours indicate bootstrap support values, from 33 (red) to 100 (green). (TIF) [file ppat.1008717.s011.tif]

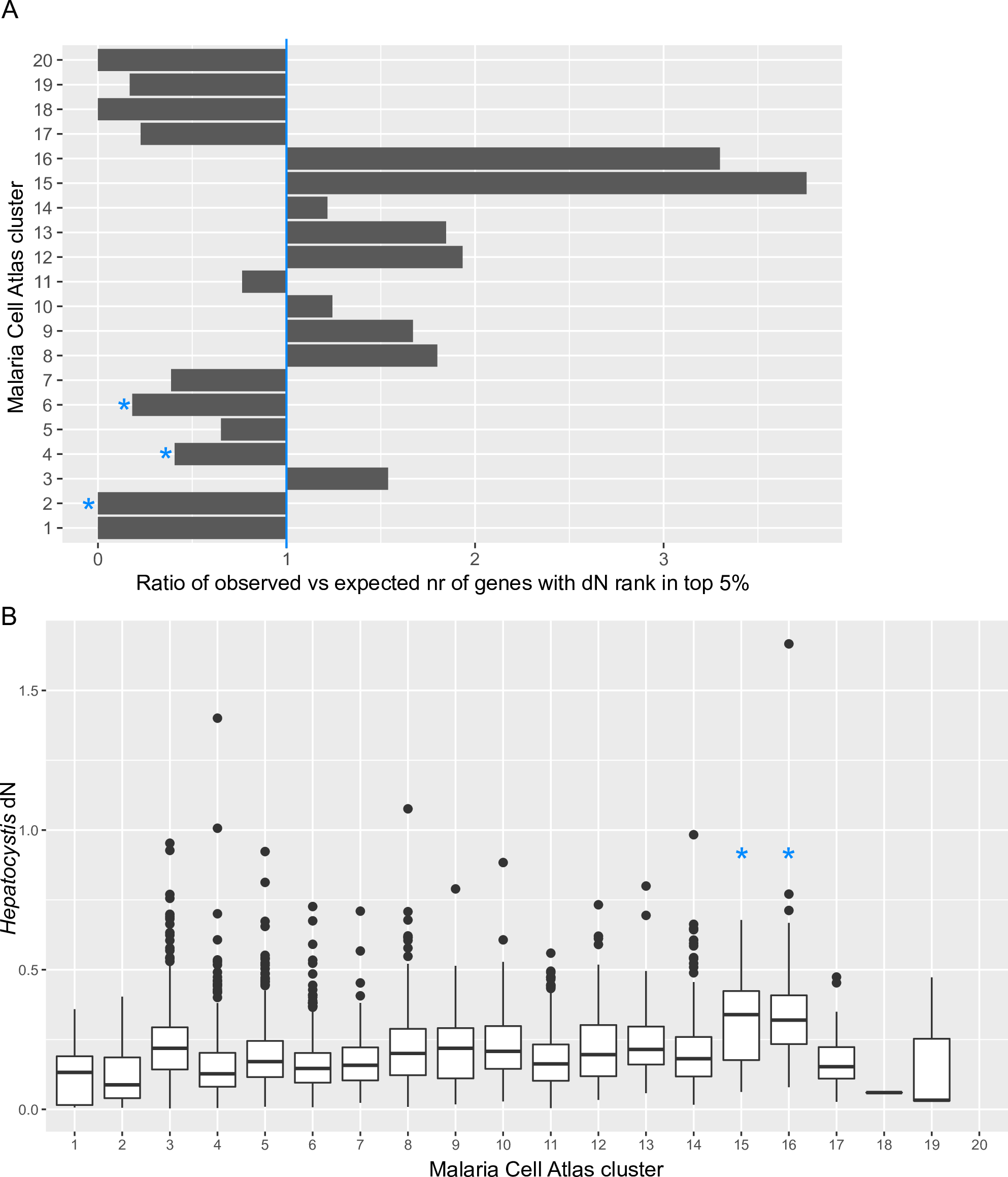

Supplement: S12 Fig — Hepatocystis dN was calculated in 3-way comparison between Hepatocystis, P. berghei ANKA and P. ovale curtisi using codeml. The Malaria Cell Atlas clusters have been described in Fig 2B in the article on Malaria Cell Atlas [20]. (A) Hepatocystis genes with dN in the top 5%: observed versus expected ratios for Malaria Cell Atlas clusters. Hepatocystis genes that correspond to Malaria Cell Atlas clusters 2, 4 and 6 have less genes with dN rank in the top 5% than expected by chance (Fisher exact test p-value < 0.05). None of the MCA clusters contain significantly more genes ranked in the top 5% of dN than expected by chance, although there is a trend towards clusters 15 and 16 having higher dN. (B) Boxplot of all Hepatocystis dN values per each Malaria Cell Atlas cluster. Distribution of values in clusters 15 and 16 differs from the rest of the clusters. Kolmogorov-Smirnov test statistics are the following. Cluster 15 vs all other clusters: D = 0.42, p-value = 1.05e-05. Cluster 16 vs all other clusters: D = 0.52, p-value = 4.50e-12. Clusters 15 and 16 combined vs all other clusters: D = 0.46, p = 2.33e-15. (TIF) [file ppat.1008717.s012.tif]
