# Supplementary material for: Genomic and transcriptomic evidence for descent from Plasmodium and loss of blood schizogony in Hepatocystis parasites from naturally infected red colobus monkeys
Source: PLoS Pathog. 2020 Aug 3;16(8):e1008717. doi: 10.1371/journal.ppat.1008717 (PMC7425995; doi:10.1371/journal.ppat.1008717)
Supplement: Attachment — Submitted filename: Hepatocystis response 200520.docx [file ppat.1008717.s017.docx]

Dear editor and reviewers,

Thank you very much for your time and your kind words about our manuscript. We found your suggestions extremely helpful and we think we have been able to improve our manuscript significantly based on them.

See below for a detailed response.

Kind regards,

Adam Reid

**Response to Reviewer 1**

**The reviewer wanted to know why we were unable to produce a more complete assembly of the *Hepatocystis* genome sequence.** In this project we used sequencing reads obtained from the blood of wild monkeys, which were sequenced for a different purpose. It might be possible to go back to these monkeys and generate more high quality, long-read sequencing data from essentially the same strain, but we considered that making the best use of currently available data without further intervening with the monkeys was worthwhile. We are currently pursuing the sequencing of further Haemosporidian genomes using long read technology, however this is not trivial given the high levels of host material in such samples. We hope in future to produce more high quality assemblies of *Hepatocystis* sp. genomes, but we believe this requires more R&D work and consider it beyond the scope of the current manuscript.

**It was suggested that we include analysis of the *Haemoproteus tartakovskyi* genome in our paper.** This was an excellent suggestion. It was something we had looked into early on and decided to leave out, but was well worth reconsidering. This species is an outgroup to *Plasmodium* and so it makes a useful anchor for phylogenetics of *Plasmodium* spp., as well as a comparator for presence/absence of genes in our analyses. Therefore, we have now included it as an outgroup in our main phylogenetic tree, showing the unambiguous placement of *Hepatocystis* sp. as a sister group to the rodent-infecting *Plasmodium* species. One focus of our manuscript is the absence of reticulocyte binding proteins (RBPs) in *Hepatocystis* sp., which we think may be related to their role in erythrocytic schizogony in *Plasmodium*. Erythrocytic schizogony is a hallmark of the *Plasmodium* genus and is thought to be absent from other Haemosporidians. We therefore sought to expand our analysis of RBPs to *Haemoproteus*. Our hypothesis being that RBPs might generally be absent from all *Haemospordia* that lack erythrocytic schizogony. The original *Hae. tartakovskyi* publication stated that there were seven orthologues of *P. falciparum* Rh1 in this genome [[1]](https://paperpile.com/c/QrMUMO/zzWvk), however we were unable to find any based on either orthoMCL analysis or reciprocal BLAST searches using only known reticulocyte binding protein sequences. It might be related to the fact that there are at least six *Plasmodium* proteins in public databases which are mislabelled as RBPs (PlasmoDB: PY03265, PKNOH_S01014400, PCOAH_00000700, PCYB_011460; GenBank: KNC36480.1, KNG75882.1). Where these relate to entries which we are able to change we are seeking to do this. We then examined a transcriptome assembly for *Hae. columbae* [[2]](https://paperpile.com/c/QrMUMO/7onZH). Here we found a fragmentary sequence with significant similarity to RBPs from *P. relictum* in a region shared across *Plasmodium* RBPs. We found no evidence for RBPs in a partial transcriptome assembly for *Leucocytozoon buteonis*, an outgroup to both *Plasmodium* and *Hemoproteus* [*[3]*](https://paperpile.com/c/QrMUMO/ptN2P). Thus, our analysis suggests that, counter to previous suggestions, RBPs may be a specific innovation of the *Plasmodium* genus, coinciding with the development of erythrocytic schizogony, albeit deriving, at least partially, from identifiable domains also present in *Hae. columbae*. These were then lost in the *Hepatocystis* lineage, coincident with the loss of erythrocytic schizogony. A wider selection of more complete genomic information will be required to flesh out this assertion. We have added this result to the manuscript to flesh out our RBP story. We have also added *Hemoproteus* *tartakovskyi* to Table 2 and compared the frequency of members of different gene families across this wider group of species, giving greater context to our analysis.

**Species of Hepatocystis have also been described from the mammalian order Artiodactyla (e.g. Hippopotamus and Tragulus). So, I recommend adding “amongst others” or also listing this mammalian host group.**

We have adjusted this in the abstract and introduction.

**The authors state: “In contrast, liver merozoites of Hepatocystis spp. are thought to commit to the development of transmission stages directly upon invading red blood cells.“ Just for clarity, I recommend to specify that the transmission stages are sexual (gametocyte) stages.**

We have clarified this point in the text

**The authors state: “At least four species of Hepatocystis are known to infect African monkeys – H. kochi, H. simiae, H. bouillezi and H. cercopitheci (6) – but with little sequence data currently linked to morphological identification, it was not possible to determine the species.” Studies of the bat-infecting Hepatocystis species have proposed that bats in Africa and Australia are rather infected with parasites that belong to Hepatocystis species-complexes than different species. Could this also apply to the primate-infecting “species”? Perhaps the authors could comment on this**.

Unfortunately, I don’t think our data can shed any light on this issue. We have seen no evidence of co-infection of multiple species/strains of *Hepatocystis* in these monkeys and very low levels of sequence diversity between infections, suggesting we observed very closely related members of the same species in all monkeys surveyed. If these parasites represent members of a species complex, then wider sampling would be required to show this.

**The authors state: “Limited sequence data are available for Hepatocystis outside of this study, however 11 nuclear genes have been sequenced for H. epomophori, a parasite of bats (2). Based on the sequence of these genes, we found that HexPt forms a sister group to H. epomophori (S5 Fig; S1 Dataset).” Limited sequence data for Hepatocystis is available and almost exclusively comprises short gene sequences for phylogenetic studies. However, it is probably still worth noting that some multiple-gene phylogenies investigated the phylogenetic placements within the Hepatocystis clade, showing that all Hepatocystis species from monkeys (from Asia and Africa) group in one monophyletic clade, sister to the Hepatocystis species that infect African bats. There is evidence that the Hepatocystis species of bats are not monophyletic and that Hepatocystis species from bat hosts of the genus Pteropus group basal to all other Hepatocystis species and therefore Hepatocystis species of monkeys might represent a derived clade. -**

This is an important point; however, we do not present data which show this, nor do we make an argument for or against it. Rather, we present data which suggest that *Hepatocystis* itself is a monophyletic group. Despite trying a couple of ways, we could not find a suitable place in the manuscript to add this.

**The authors state: “In vivo transcriptome data supports a lack of erythrocytic schizogony. Transcriptome sequencing of blood samples from 29 individuals was performed as part of the red colobus monkey genome sequencing project (12). We found evidence that each of these individuals was infected with the same species of Hepatocystis as found in the genomic reads, consistent with high prevalence of this parasite in Kibale red colobus monkeys as previously reported (6)." Mixed haplotype infections have been reported/investigated by Thurber et al. How did the authors deal with the mixed haplotype infections in their samples? Did the sequences that were detected within the monkey genome sequences show hints of a mixed infection?**

In Figure 4 of the Thurber et al. paper (2013), some of the present authors showed that colobus can be infected with either lineage 2 or 3 or both, and that either can be dominant.  However, they are not equal.  In co-infected red colobus, lineage 2 is dominant in all but one monkey, and in that monkey lineages 2 and 3 are equally abundant.  So, one possibility is that in the present study we picked up only dominant lineage 2.  These lineages were defined using cytochrome b and it is possible that, across the genome, the two lineages are really very closely related.  If you look at Thurber et al. Figure 3, the difference between lineages 2 and 3 is only 1-2% in cytb, so it may be 10- to 30-fold lower in the nuclear genome.

Indeed, what we found in the RNA-seq samples was a low rate of SNPs across the genome and no evidence for mixed infections. We performed SNP calling in RNA-Seq libraries and it showed low diversity among the *Hepatocystis* samples (Figure 3A in the submitted manuscript). We have now also generated a phylogenetic tree of the RNA-seq SNPs which shows that the RNA-seq samples differ from one another by similar distances, rather than belonging to distinct subgroups, showing that there is no clustering of the samples and no evidence for distinct sequences between infections (Response Figure 1).

**Response Figure 1. Phylogenetic tree of SNPs in *Hepatocystis* RNA-Seq samples**

This was based on variant calling with GATK 4.0.3.0 [[4]](https://paperpile.com/c/QrMUMO/bMktW), plotted using SNPRelate 1.16.0 [[5]](https://paperpile.com/c/QrMUMO/hNwb8) and ape 5.3 [[6]](https://paperpile.com/c/QrMUMO/mm0Hl) R packages. Samples SAMN07757853, SAMN07757863, SAMN07757870 and SAMN07757873 were excluded from the figure due to their low expression of *Hepatocystis* genes.

When assembling *Hepatocystis* RNA-Seq and DNA sequencing data, a subset of contigs appeared as duplicated (with less than 3% difference from another contig) in the raw output from the assemblers. Some of these duplicated contigs could possibly represent different haplotypes in the sequenced sample. However, in our experience, duplicated contigs also occur when assembling the genomes of cultured *Plasmodium* strains, where the DNA has been extracted from a haploid stage. This is presumably due to mismatches and indels in reads caused by sequencing errors. In RNA-Seq assemblies, this is further complicated by different isoforms of the same transcript being output as separate contigs. Overall, it looks like either all the monkeys in the study are infected with the same strain of *Hepatocystis*, or if there is more than one *Hepatocystis* strain in the dataset, these strains are very closely related.

We also looked for signs of mixed infection with *Plasmodium* in the samples but did not find any (e.g. only *Hepatocystis* cytochrome b and no *Plasmodium* cytochrome b was found in all RNA-Seq samples).

**The manuscript contains a few spelling errors/typos. In several cases, “spp.” is erroneously written italics (e.g. abstract, results) and “Vinckei” should be “Vinckeia”.**

These errors have been amended

**Have permits and agreements been expanded to allow use of DNA samples for parasite analysis in addition to the originally planned analysis of the monkey genome? (Nagoya/CBD)**

All original permits and agreements were obtained for research combining both primate genetics and pathogen identification/characterization.  Thus, the original permits already cover all uses of samples and data described in the manuscript.

**Response to Reviewer 2**

**I first want to highlight my perception of the special character of the manuscript as an asset: I consider the (very successful) re-use of host-genome "contamination" for parasite research a central aspect of the study. In my opinion this could be more clearly stated as not only viable but attractive for parasitologists working with genomic methods. The first sentence of the discussion could e.g. easily include a statement on data provenance. I also find the  first sentence of the methods could be improved (missing "a" before "project", instead of "a different project", rather "a project with the aim to generate" or similar). In my opinion the manuscript could be generally more pronounced on this point.**

We agree that the re-use of the data is something we should make more of in the manuscript. We have added/altered text to make this clearer at the start of the results, methods and discussion sections.

**Taxonomy: The data is from only one species of the genus Hepatocystis. This is made clear in the context of the whole manuscript (esp. in the introduction "thus classified the parasite as Hepatocystis sp. ex Piliocolobus tephrosceles (HexPt; NCBI Taxonomy ID: 2600580)". In my opinion, nevertheless, Hepatocystis _sp._ should be used to refer to a - for the purpose of this manuscript undetermined - species in this genus throughout the manuscript (I'd prefer this over the more technical HexPt, but this is a matter of taste).**

We had some doubts about the use of HexPt and have altered this throughout the manuscript to the less intrusive *Hepatocystis* sp.

**Fig 1 A. This figure and the corresponding analysis could include additional information on the coverage profiles for the contigs/scaffolds with the different sequence similarity and GC (see e.g. "blobtools"). The signal of (low) GC content is expectedly strong for a Plasmodium sp./Hepatocystis sp. data set, it would be great to have sequencing coverage as a second visually distinguishing feature. This could also be (an) additional panel(s) for different sequencing libraries.**

We show below the results of including coverage as a factor in picking apart contigs (Response Figure 2). We don’t think that it adds very much in this case and we have not used it to separate our contigs. Therefore, we have decided not to incorporate these into the manuscript.

Here are the plots with the coverage of the mixed-species DNA reads when mapped to the *Piliocolobus* assembly (ASM277652v1). All Illumina HiSeq4000 and HiSeq X reads (containing a mix of *Hepatocystis* and *Piliocolobus* sequences) were mapped to the *Piliocolobus* assembly ASM277652v1 with Minimap2 2.17-r941 [[7]](https://paperpile.com/c/QrMUMO/AsonI). Chromium bar codes were removed from all the reads before mapping (using chromium_reads_remove_barcodes.cpp in our GitHub repository). Coverage values of each scaffold were derived using SAMtools depth 1.9 [[8]](https://paperpile.com/c/QrMUMO/6UMMV) and plotted in R. The average coverage and the average GC of every scaffold were plotted as a scatter plot. Colours of the dots correspond to the results of identifying the contigs with sequence similarity-based methods (BLAST, Diamond and competitive mapping with Minimap2).

A

B

C

**Response Figure 2.** Using coverage as a factor to identify contigs from different species. A. HiSeq 4000 coverage and GC%. B. HiSeq X coverage and GC%. C. Coverage sum of HiSeq X and HiSeq 4000 reads vs GC%

**Fig 1 B. The presented colour legend should be changed/expanded to separate geographical information from host species information. Information on the latter should also be included for Plasmodium species, like in figure 2 (ideally using the same symbols).**

This is an excellent suggestion and we have changed the representation of these data

**Fig 2. In my opinion the phylogenetic tree - transporting the core message of the whole figure - should attract more attention in this. The "figure columns" "ap2 protein" and "tRNA" take (together) a similar amount of space as the tree but show relatively little variation, hence contain hardly any information.**

We have removed several columns from this figure, as suggested, to improve the presentation of the tree.

**I am missing a figure for the paragraph "Missing orthologues tend to be involved in erythrocytic schizogony". This lacks representation in both main text figures or tables. Plotting expected/observed ratios for the number of orthologues found (like for SNPs in figure S11, but see comment below!) could potentially help to visualise missing or surplus orthologues in MCA clusters. Using this as an anchor (with Hepatocystis sp. data), visualisation of "Cluster 4" and "Cluster 10" from MCA relying on P.berghei data (Figure S10) could be included as additional panels. This might help to introduce the interpretation of MCA clusters for the present manuscript generally. MCA clusters play an important role as "custom annotation" in two parts of the analysis: enrichment of genes missing in Hepatocystis sp. and enrichment of high-dN gens in those clusters. It could be nice to introduce MCA more visually this way as part of a main manuscript figure!**

This is a great suggestion. We had been looking for a good way to add a redrawing of the clusters from the MCA paper, without which we thought it difficult for the reader to really understand the different clusters. We have redrawn Figure 2B from the MCA paper, using the supplementary data and added the observed/expected ratios used in calculating the clusters with unexpectedly high levels of missingness in *Hepatocystis*. This has been added as a new Figure 3 in the main manuscript.

**Fig 3. The caption of this figure only refers to figure panel B (and somewhat to C), but not to A. For A, I am also missing the information (in the legend) that the SNPs are called relative to the genome assembly(?). I am wondering whether A could be a figure on its own (SNPs in the transcriptome), maybe with an additional panel presenting the enrichment of SNPs in the MCA clusters (currently S11)?**

We have addressed the problem with the caption for Figure 3 (now Figure 4), adjusting it from “Deconvolution of *Hepatocystis* sp. *in vivo* RNA-seq data supports a lack of erythrocytic schizogony and a variable sex ratio“, to “*Hepatocystis* sp. *in vivo* RNA-seq data supports a lack of erythrocytic schizogony and a variable sex ratio”. We have added information for panel A, stating that the genome was used as reference. We think that panel A sits best with the rest of the figure in terms of the narrative of the paper. Figure S11 addresses enrichment of high dN/dS values across species rather than SNPs within species, so we think that it is not appropriate to put this with Figure 4 (now 4 A).

**Btw. Fig S11 A should make more clear that a ratio of 1 is the null expectation: rotation by 90° and plotting deviations from 1 (as bars or lines) would help to make this more obvious.**

Fig S11A has been adjusted as suggested.

**Table 2 should, in my opinion, contain context for the expected size of gene families. Listing the family size from Plasmodium species (e.g. P. berghei, P. vivax and P. falciparum; just like in the genome comparisons of Table 1) would add such context. Also: "Frequency of members" sounds a bit too technical to me, isn't this simply the "gene family size"?**

Agreed. This suggestion massively improves the table. We have also added *Haemoproteus* based on suggestions from reviewer 1.

**References**

1.	[Bensch S, Canbäck B, DeBarry JD, Johansson T, Hellgren O, Kissinger JC, et al. The Genome of Haemoproteus tartakovskyi and Its Relationship to Human Malaria Parasites. Genome Biol Evol. 2016;8: 1361–1373.](http://paperpile.com/b/QrMUMO/zzWvk)

2.	[Toscani Field J, Weinberg J, Bensch S, Matta NE, Valkiūnas G, Sehgal RNM. Delineation of the Genera Haemoproteus and Plasmodium Using RNA-Seq and Multi-gene Phylogenetics. J Mol Evol. 2018;86: 646–654.](http://paperpile.com/b/QrMUMO/7onZH)

3.	[Pauli M, Chakarov N, Rupp O, Kalinowski J, Goesmann A, Sorenson MD, et al. De novo assembly of the dual transcriptomes of a polymorphic raptor species and its malarial parasite. BMC Genomics. 2015;16: 1038.](http://paperpile.com/b/QrMUMO/ptN2P)

4.	[McKenna A, Hanna M, Banks E, Sivachenko A, Cibulskis K, Kernytsky A, et al. The Genome Analysis Toolkit: a MapReduce framework for analyzing next-generation DNA sequencing data. Genome Res. 2010;20: 1297–1303.](http://paperpile.com/b/QrMUMO/bMktW)

5.	[Zheng X, Levine D, Shen J, Gogarten SM, Laurie C, Weir BS. A high-performance computing toolset for relatedness and principal component analysis of SNP data. Bioinformatics. 2012;28: 3326–3328.](http://paperpile.com/b/QrMUMO/hNwb8)

6.	[Paradis E, Claude J, Strimmer K. APE: Analyses of Phylogenetics and Evolution in R language. Bioinformatics. 2004;20: 289–290.](http://paperpile.com/b/QrMUMO/mm0Hl)

7.	[Li H. Minimap2: pairwise alignment for nucleotide sequences. Bioinformatics. 2018;34: 3094–3100.](http://paperpile.com/b/QrMUMO/AsonI)

8.	[Li H, Handsaker B, Wysoker A, Fennell T, Ruan J, Homer N, et al. The Sequence Alignment/Map format and SAMtools. Bioinformatics. 2009;25: 2078–2079.](http://paperpile.com/b/QrMUMO/6UMMV)
